# Intravenous sedation in dental implant surgeries: A systematic review of hemodynamic effects

**DOI:** 10.15171/japid.2019.009

**Published:** 2019-12-18

**Authors:** Reza Pourabbas, Nazgol Ghahramani, Mehrnoosh Sadighi, Hassan Soleimanpour, Mohammad-Salar Hosseini, Fatemeh Pournaghi Azar

**Affiliations:** ^1^Department of Periodontics, Faculty of Dentistry, Tabriz University of Medical Sciences, Tabriz, Iran; ^2^Student, Faculty of Dentistry, Tabriz University of Medical Sciences, Tabriz, Iran; ^3^Aging Research Institute, Tabriz University of Medical Sciences, Tabriz, Iran; ^4^Student Research Committee, Faculty of Dentistry, Tabriz University of Medical Sciences, Tabriz, Iran; ^5^Department of Esthetics and Restorative Dentistry, Faculty of Dentistry, Tabriz University of Medical Sciences, Tabriz, Iran

**Keywords:** Dental implants, hemodynamic effects, intravenous sedation

## Abstract

**Background:**

The main objective of thissystematic review wasto identify the hemodynamic effects of intravenous sedatives used in dental implant surgeries.

**Methods:**

Embase, PubMed, ProQuest, Scopus, Ovid, and Cochrane databases were searched with no limitations. Of 59 studies obtained, 50 studies were excluded due to incompatibility with the subject. The remaining studies were reviewed in full text and assessed for the risk of bias individually. The included studies were reviewed by the research team, and the necessary data were extracted.

**Results:**

Four studies were finally included. Two of the studies compared local anesthesia and intravenous sedation, while the other two compared the consequences of different types of intravenous sedation. By comparing the hemodynamic effects, the systolic and diastolic blood pressure and the heart rate data were collated. Midazolam was the most frequently used intravenous sedative, and Dexmedetomidine affected hemodynamics the most.

**Conclusion:**

Intravenous sedation leads to decreased heart rate and blood pressure. Better hemodynamic outcomes improve the patients’ cooperation by decreasing stress and anxiety. Dexmedetomidine seems to be the first choice for intravenous sedation.

## Introduction

Like every other dental surgery, Dental implant surgery could also result in anxiety, pain, systemic complications, and even life-threatening conditions.^1^ These situations might affect the results of the surgery, patients’ satisfaction, and even the recovery duration.^2,3^ Poor outcomes of the surgery and low satisfaction affect patients’ further cooperation. As most of the dental procedures are inevitable, numerous supportive solutions – like different sedation methods – have been developed over time.

Based on the type, duration, and workload of the procedure, a variety of sedation methods are available, ranging from the inhalers (commonly known as the laughing gas) to intravenous (IV) sedatives.^4,5^ Although most of the straightforward procedures take place with the administration of local anesthetics, stronger anesthetics with broader effects are needed to reduce pain and increase the duration of sedation in lengthy dental procedures.^6^ Intravenous sedation is an appropriate and efficient method, which is useful in decreasing pain and anxiety in dental implant surgeries.^7,8^ A variety of intravenous sedation methods are available, and different methods might result in different outcomes. The proper intravenous sedative is usually picked according to the procedure and based on the expertise.^9^ Even though many differences have been observed between these IV sedation methods previously, when these methods are compared, no decisive standpoint is available. This lack of information makes the drug choice more difficult, as most of the consequences of a wrong choice – like cardiovascular complications – could be life-threatening.^10^

Therefore, the current study was conducted to fill the gap by reviewing the characteristics of previously studied intravenous sedation methods and their effects on hemodynamics.

## Methods

### Study Design

A comprehensive systematic literature review of research databases was conducted through the Embase, PubMed, ProQuest, Scopus, Ovid, and Cochrane from 1990 up to August 2019. The search strategy included a combination of Mesh and free keywords. Also, for more precise results, a manual search was performed among the references of the collected articles. For the study selection, the PICO (Population, Intervention [or exposure], Comparison, and Outcome) framework was used to clarify the search.

Population: Patients with implant surgeries (≥18 years of age, healthy patients participating in an RCT) who were in the American Society of Anesthesiologists’ (ASA) group I or II

Intervention: IVCS

Comparison: LA

Outcome: Hemodynamic effects

**Figure 1:**
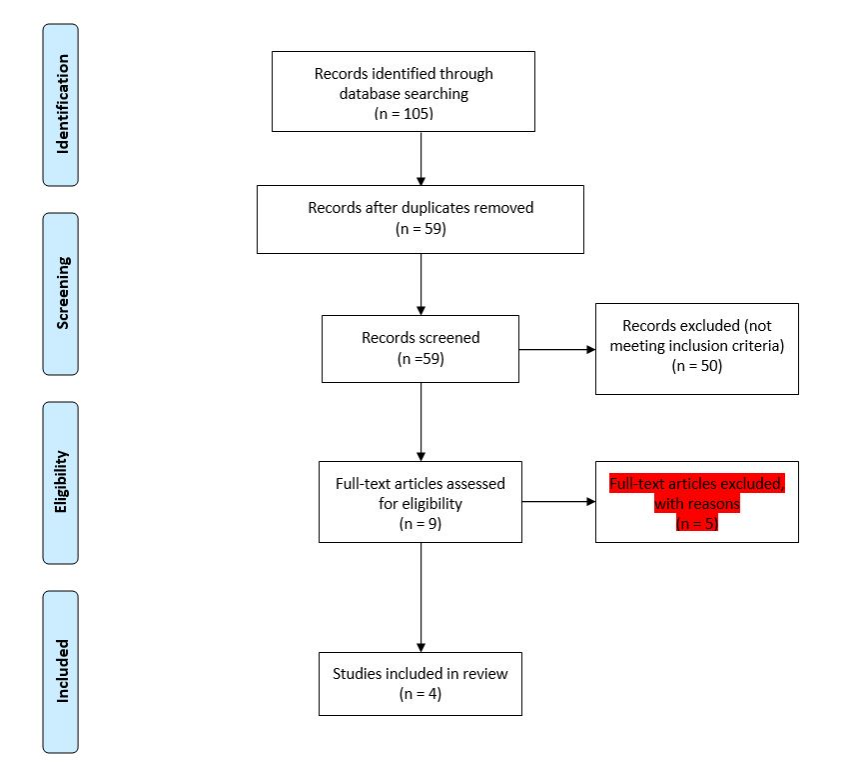
PRISMA flow-diagram of the study.

The search, selection, and assessment processes were performed in four steps, conforming to the PRISMA flow diagram shown in Figure 1. The steps were (i) systematic literature search, (ii) removal of duplicates, (iii) identification of potentially relevant articles based on the title and abstract, and (iv) full-text screening. The first and second authors, assisted by a librarian, performed the search string for the electronic search. For more precise results, a manual search was performed in the references of the collected articles. Also, the research team contacted the study authors to obtain additional information if necessary. The first author screened titles and abstracts, and the first and second authors read the full texts of the remaining articles separately for inclusion or exclusion in the review. The selected articles were appraised based on Cochrane’s tool for assessing the risk of bias in randomized trials.^11^ The articles were appraised by two members of the research team, and the points of disagreement were referred to a third arbitrator. The risk of bias was evaluated by Cochrane risk of bias checklist by RP and MS. In case of disagreement, the assessors reached an agreement by discussing the differences. The included studies were reviewed, and the necessary data were extracted by two reviewers independently using the standardized data extraction tool. The results were reported as mean ± SD. The study design is summarized in Figure 1. The inclusion criteria for the studies were as follows:

Randomized control trials (RCT) with patients who had dental implant surgeries (≥18 years of age, healthy patients) and were in the American Society of Anesthesiologists’ (ASA) group I or II, with the intervention group of IVCS being included. RCTs published in languages other than English were excluded. Duplicate data, low-quality RCTs, gray literature, and letters to editors were excluded from this systematic review. Quality assessment and risk of bias for each RCT were assessed according to the JBI checklist.

## Results

The search results are summarized in Figure 1, according to the PRISMA method.^12^ Of 59 collected articles, 50 were excluded due to a lack of conformity with the subject. After reviewing the full texts of the remaining nine articles, four studies were selected. Overall, the eligible studies included 412 patients, consisting of 224 females (54.4%) and 188 males (45.6%). Midazolam was used in all the studies as the main or primary IV sedative. Two of the studies examined the effectiveness of IV sedation in comparison to the local anesthesia,^13,14^ while the other two studies aimed to determine the effects of different IV sedatives.^15,16^ Systolic and diastolic blood pressure (BP) and heart rate (HR) are reported in Table 1, along with the general characteristics of each study. For studies reporting blood pressure in different phases, the sedation phase was considered in comparisons.

**Table 1:** Data extraction of the included studies

**#REF!**	**year**	**Total sample size**	**group**		**sample size**		**Sex**		**Heart rate (bpm)**		**Systolic blood pressure (mmHg)**		**Diastolic blood pressure (mmHg)**	
**Fan, T. W., et al (2012)**	2012	60	a:dexmedetomidine	b:midazolam	a:30	b:30	a:female:11	b:female:7	a:max:80 (11)	b:max:85 (14)	a:max:126 (12)	b:max:129 (14)	a:max:79 (9)	a:max:81 (11)
			4 g/ml	0.2 mg/ml			male:19	male:23	min:59m (10)	min:67 (13)	min:99(10)	min:104(12)	min:54(7)	min:60(9)
**Taguchi et al (2011).**	2011	255	a: Midazolam + propofol + Local Anesthesia	b: Local Anesthesia	a:123	b:132	Female: 140	male:115	a:mean:80	b:mean:92	a:mean:122	b:Mean:163	a:mean:79	b:mean:106
**Win, N. N., et al (2005)**	2005	30	a:Propofol 1 g/mL	b:midazolam 0.5 mg	a:15	b:15	a:female:10	b:female:9	a:mean:73	b:82	a:104	b:112	a:63	b:67
							male:5	male:6	SD:5	7	10	14	8	9
**Juodzbalys, G., et al**	2005	87	a:midazolam 0.1 mg/kg	b:articaine 4%	a:67	b:20	a:female:37	b:female:9	a:mean:80	b:92	a:122	b:163	a:79	b:106
			60 mg of ketorolac	and epinephrine			male:30	male:11						

*Data are represented as Mean (SD)

## Discussion

In the present study, the hemodynamic effects of intravenous sedation in oral surgeries were reviewed. The results demonstrated that IV sedation methods could lead to significantly lower blood pressure and heart rate. Among the four included studies, Midazolam, Dexmedetomidine, and Propofol were the main points of interest. Two studies compared the local anesthesia versus the combination of the local anesthesia with IV methods.^13,14^ The other two studies aimed to compare the effects of the most common IV methods.^15,16^ Midazolam and local anesthesia were a part of the sedation choice in all the included studies, and local anesthesia was applied, using either lidocaine or articaine.

### Intravenous Sedation vs.LocalAnesthesia

The most critical role of sedation in oral surgeries is to reduce the patients’ pain and anxiety.^8,17^ This could result in two important outcomes: better cooperation from patients and higher patients’ satisfaction.^16^ Two studies showed that IV sedation methods had better hemodynamic effects in comparison with the simple local anesthesia, and the heart rate decreased by 12 bpm (P<0.05). As the heart rate is a direct manifestation of patients’ fear and anxiety, the significant decrease in heart rate could be interpreted as a sign of stress reduction.^18^ On the other hand, in comparison with the local anesthesia, the decrease in both systolic and diastolic blood pressure is evident in patients receiving IV sedatives. The discussed effects of IV sedation on hemodynamics have been studied previously. Many studies have demonstrated the fact that oral surgery under IV sedation with local anesthesia results in much less stress and significantly more stable hemodynamics, rather than both general and local anesthesia.^10,19,20^ Also, studies conducted on the effects of different sedatives in surgeries of other body parts show the same results, as the hemodynamic variables are close to the baseline.^21-23^

The underlying mechanisms for these effects are described as decreased epinephrine and norepinephrine levels of plasma (high-affinity binding to α2-adrenoceptors), resulting in decreased sympathetic outflow.^24,25^ Therefore, hypotension and decreased heart rate are observed. Effects on the parasympathetic nervous system are another suggested mechanism, mostly considered for Propofol.^15,26^

### Which Intravenous Sedative Is Preferable?

Different types of intravenous sedatives have different outcomes. A study by Fan et al^16^ showed that in comparison with Midazolam, Dexmedetomidine injection resulted in a significantly lower heart rate and blood pressure. In addition, a study by Win et al^15^ demonstrated that under intravenous injection of Propofol, the patients had more stable hemodynamics. Several previous studies have also discussed and compared the effectiveness of different intravenous sedatives, showing a broad spectrum of efficacy.^27-30^ According to the results on the hemodynamic properties, Dexmedetomidine is the most effective choice, being more effective than Propofol and Midazolam. Midazolam is known to be less effective in hemodynamics. Some studies have even shown no significant changes in heart rate and blood pressure regarding the administration of Midazolam.^31^

### Other Systemic Effects of Intravenous Sedation

In addition to improved hemodynamics, reduced bleeding, better safety profile, decreased retrograde amnesia, improved patient cooperation, stable respiratory conditions, and higher patients’ satisfaction are other known advantages of IV sedation.^5,32,33^ Apart from the benefits of IV sedatives, there are very few disadvantages known, only including a longer duration of surgery and discomfort on injection. Also, bradycardia is a discussed adverse effect of IV sedatives, mainly Dexmedetomidine.^24^ Further studies are needed to discover more evidence on the safety, efficacy, and underlying mechanisms involved in the different outcomes of various intravenous sedatives.

## Conclusion

Intravenous sedation seems to be more heart-friendly by considering better hemodynamic outcomes. Decreased heart rate and blood pressure also result in less stress and better patient cooperation. Considering the intravenous sedatives, Dexmedetomidine, Propofol, and Midazolam had better outcomes, respectively.

## Authors’ Contributions

The study was planned by RP and NG. Data collection was carried out by NG and MS; statistical analyses and interpretation of data were carried out by FPA and RP. The manuscript was prepared by MSH and HS and revised by MS. All the authors have read and approved the final manuscript for submission.

## Competing Interests

The authors declare that they have no competing interests with regards to authorship and/or publication of this paper.

## Ethics Approval

The study protocol was approved by the Ethics Committee in Medical Research, Tabriz University of Medical Sciences under the code IR.TBZMED.VCR.REC.1397.447.
